# Comparison between MRI-negative and positive results and the predictors for a poor prognosis in patients with idiopathic acute transverse myelitis

**DOI:** 10.1186/s12883-024-03738-5

**Published:** 2024-07-01

**Authors:** Yu Zhou, Qianxi Chen, Weiming Gan, Xiuwen Lin, Bo Wang, Meihong Zhou, Xiaomu Wu, Daojun Hong, Hao Chen

**Affiliations:** Department of Neurology, The First Affiliated Hospital of Nanchang University, Nanchang, Jiangxi Province 330006 China

**Keywords:** Idiopathic acute transverse myelitis, MRI-negative, Poor outcome, Predictive factors

## Abstract

**Background:**

Idiopathic acute transverse myelitis (IATM) is a focal inflammatory disorder of the spinal cord that results in motor, sensory, and autonomic dysfunction. However, the comparative analysis of MRI-negative and MRI-positive in IATM patients were rarely reported.

**Objectives:**

The purpose of this study was to compare MRI-negative with MRI-positive groups in IATM patients, analyze the predictors for a poor prognosis, thus explore the relationship between MRI-negative and prognosis.

**Methods:**

We selected 132 patients with first-attack IATM at the First Affiliated Hospital of Nanchang University from May 2018 to May 2022. Patients were divided into MRI-positive and MRI-negative group according to whether there were responsible spinal MRI lesions, and good prognosis and poor prognosis based on whether the EDSS score ≥ 4 at follow-up. The predictive factors of poor prognosis in IATM patients was analyzed by logistic regression models.

**Results:**

Of the 132 patients, 107 first-attack patients who fulfilled the criteria for IATM were included in the study. We showed that 43 (40%) patients had a negative spinal cord MRI, while 27 (25%) patients were identified as having a poor prognosis (EDSS score at follow-up ≥ 4). Compared with MRI-negative patients, the MRI-positive group was more likely to have back/neck pain, spinal cord shock and poor prognosis, and the EDSS score at follow-up was higher. We also identified three risk factors for a poor outcome: absence of second-line therapies, high EDSS score at nadir and a positive MRI result.

**Conclusions:**

Compared with MRI-negative group, MRI-positive patients were more likely to have back/neck pain, spinal cord shock and poor prognosis, with a higher EDSS score at follow-up. The absence of second-line therapies, high EDSS score at nadir, and a positive MRI were risk factors for poor outcomes in patients with first-attack IATM. MRI-negative patients may have better prognosis, an active second-line immunotherapy for IATM patients may improve clinical outcome.

## Introduction

Idiopathic acute transverse myelitis (IATM) is a subgroup of inflammatory myelopathies of undetermined etiology, that present as acute spinal cord dysfunction. The disease is characterized by a triad of motor, sensory and/or autonomic abnormalities [1]. It is also considered a diagnostic dilemma in current clinical practice, given that a variety of underlying pathogenic factors need to be excluded. These include inflammatory conditions such as multiple sclerosis (MS), neuromyelitis optica spectrum disorders (NMOSD), acute disseminated encephalomyelitis (ADEM), the clinically isolated syndrome (CIS), myelin oligodendrocyte glycoprotein associated disorders (MOGAD), glial fibrillary acidic protein astrocytopathy (GFAP), in addition, compressive, neoplastic, vascular, metabolic, or infectious etiology are also need to be ruled out [2]. In combination with cerebrospinal fluid (CSF) analysis, spinal cord magnetic resonance imaging (MRI) is a particularly important tool for diagnosing IATM, with a relatively large proportion of IATM patients having normal spinal cord neuroimaging [3]. In cases in which MRI fails to identify lesions, spinal cord inflammation is confirmed either by gadolinium enhancement or by CSF evaluation [i.e., pleocytosis or elevated IgG index or oligoclonal bands (OB)] [4].

The diagnosis of IATM is based on the criteria of the 2002 Transverse Myelitis Consortium Working Group (TMCWG) and is based on clinical, laboratory, and neuroimaging evaluations [1]. It has been 21 years since the IATM criteria were published, although newly discovered CNS demyelinating antibodies such as aquaporin-4 immunoglobulin G (AQP4-IgG), MOG-IgG, and GFAP-IgG] are now considered to be specific biomarkers for determining the etiology of myelitis, previously thought to be idiopathic. In addition, advancements in neuroimaging have contributed to identifying alternative specific diagnoses outside of the traditional diagnostic criteria for IATM. Therefore, a contemporary reassessment of IATM characteristics is necessary [5]. When determining the radiological features of patients with IATM, previous studies have focused mainly on the site, distribution and extent of the lesions, the number of segments involved, and the presence of gadolinium contrast enhancement. However, to date, little is known about MRI-negative IATM. IATM accompanied by a negative spinal cord MRI may lead to diagnostic uncertainty and delay in treatment [3, 6]. The prognosis of patients with IATM varies, with approximately one-third of patients having a good outcome, one-third having moderate disability, and one-third left with severe disabilities [7]. Despite rapid progress in MRI technology and the detection of autoimmune biomarkers that provide not only a timely diagnosis but also the introduction of adequate immunosuppressive therapy, the prognostic implications of a negative MRI and poor prognosis in IATM patients remain uncertain.

In this study, we compared the MRI-negative with MRI-positive in patients with first-attack IATM, which including the clinical characteristics, demographics, laboratory examinations, treatment, prognosis, and relapse. In addition, predictors for a poor prognosis were also identified in our study.

## Materials and methods

### Patient selection

**Fig. 1:**
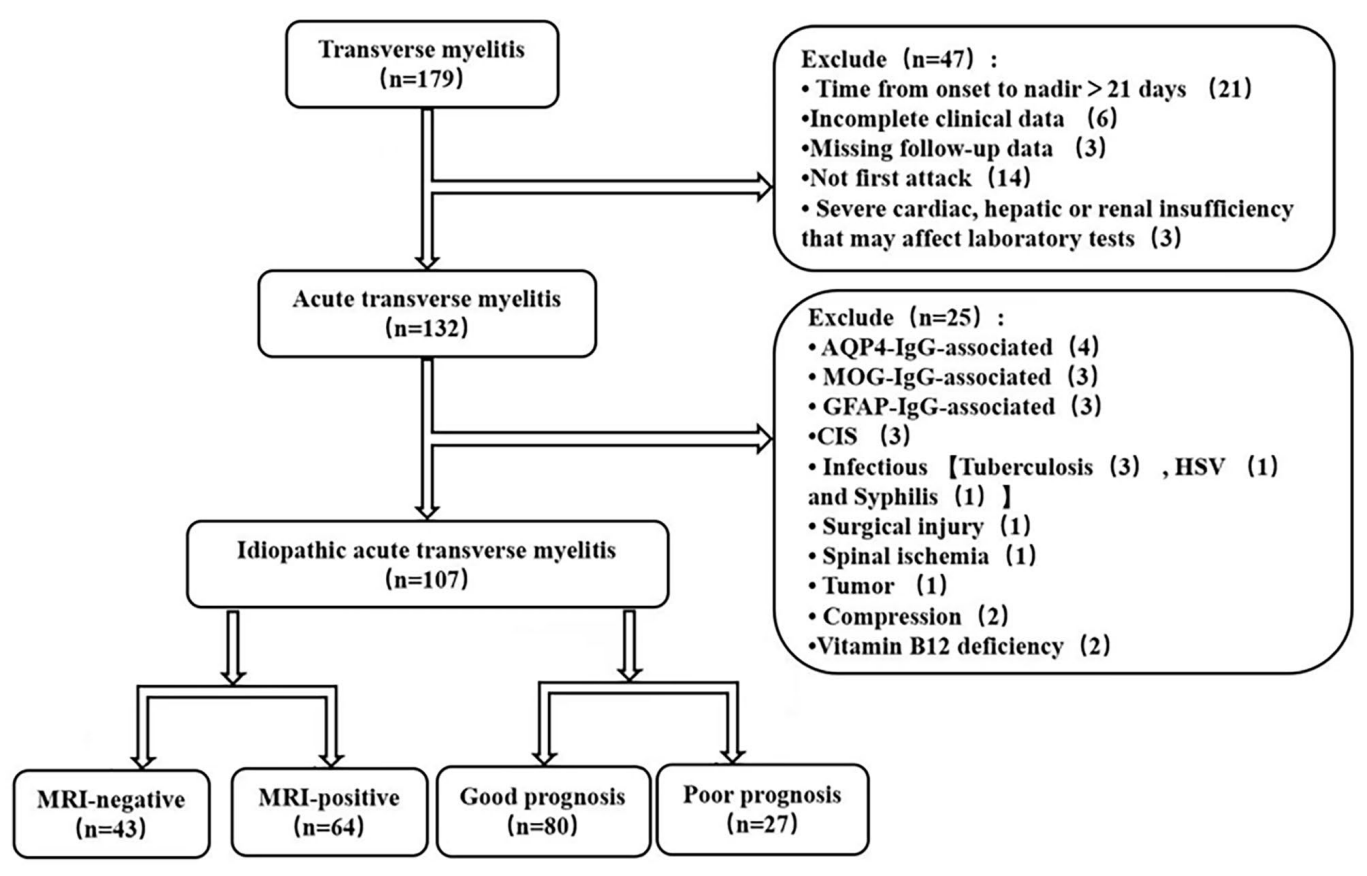
Patients identified with IATM selection flow chart

We retrospectively collected data from IATM patients admitted to the First Affiliated Hospital of Nanchang University from May 2018 to May 2022. Figure 1 shows the detailed selection process. Enrolled patients who met the following TMCWG diagnostic criteria were considered to definitely have IATM: (1) An acute first episode of sensory, motor, or autonomic dysfunction (time from onset of symptoms to nadir between 4 h and 21 days) attributed to a spinal cord lesion; (2) bilateral signs or symptoms but not necessarily symmetrical; (3) a clearly defined sensory level; and (4) evidence of spinal cord inflammation including either CSF pleocytosis, an elevated IgG index, intrathecal oligoclonal bands, or gadolinium enhancement in a spinal cord MRI. Patients with the following conditions were excluded from the study: (1) a lack of complete clinical or follow-up data; (2) severe cardiac, hepatic, or renal insufficiency that may affect laboratory tests; (3) any other known cause including either inflammatory, demyelinating (AQP4-IgG-associated, MOG-IgG-associated, or GFAP-IgG-associated CIS), vascular (spinal ischemia), infectious (tuberculosis, HSV or syphilis), or metabolic (vitamin B12 deficiency) conditions, compressive or surgical trauma, or conditions with a radioactive or paraneoplastic etiology; and (4) not first attack. The following initial examinations had been carried out in all the patients: standard blood routine tests, blood biochemical values, serum electrolytes, blood coagulation function, thyroid hormones, serum tumor markers, folic acid, vitamin B12, viral (Herpes virus groups, human immunodeficiency virus, hepatitis B and C), bacteriological (syphilis, tuberculosis) assessments, CSF analysis, CNS demyelinating antibodies testing, brain and spinal cord MRI. The breadth of rest evaluation varied as deemed clinically appropriate to rule out other differential diagnoses under TMCW guidelines, and included the following: copper, ceruloplasmin, lyme disease serology, serological parasite testing, autoimmune assays (antinuclear antibodies, anti-neutrophil cytoplasmic antibodies, antiphospholipid antibodies, protein electrophoresis), paraneoplastic autoantibody evaluation, CT-scans, PET-CT, color ultrasound, EMG and biopsies, spinal magnetic resonance angiography, digital subtraction angiography, visual evoked potential, and ophthalmological examination. The study was a retrospective, observational, single-center design and was approved by the Medical Ethics Committee of the First Affiliated Hospital of Nanchang University (approval ID: 2023CDYFYYLK(04–018).

### Data collection

Basic clinical and demographic information of the enrolled patients was collected, including age at onset, sex, clinical symptoms, laboratory tests (blood cell, blood biochemistry, and coagulation function), CSF analysis (WBC count, protein level, oligoclonal bands, and IgG index), MRI examination, treatments, time from onset to first MRI and to the maximum deficit, length of hospital stays, duration of follow-up, EDSS at nadir and follow-up, intensive care unit (ICU) admission, complications, prognosis, and relapse.

### Treatment and relapse

The patients received different therapeutic regimens according to their clinical condition and financial situation. Patients with a good financial background or those with a poor response to the initial administration of intravenous corticosteroids (i.e., methylprednisolone or dexamethasone), received second-line treatment that included intravenous immunoglobulins (IVIg) or plasma exchange (PLEX)]. A relapse was defined as any new sign or worsening of central nervous system (CNS) symptoms, and was supported by clinical examination or radiologic lesions.

### Radiological examination

The MRI scans were performed using 3.0T magnets. The patients were not imaged according to a standardized protocol due to the retrospective nature of the study. However, all the spinal cord MRIs included axial and sagittal images of the spinal cord obtained by T1 with or without gadolinium enhancement and T2 sequences. The characteristics of the neuroimaging were assessed independently by two radiologists who were blinded to the clinical information of the patients. The location of the lesions was identified as cervical, thoracic, lumbar, or sacral spine.

### Assessment of function

The severity of the disease was assessed at nadir by the widely used Expanded Disability Status Scale (EDSS) [8]. A poor outcome at the latest follow-up (at least one year after discharge) was defined as an EDSS score ≥ 4, while a good outcome was defined as an EDSS score < 4. Disability was classified into three categories: mild (EDSS between 0 and 3.5), moderate (EDSS between 4 and 7) and severe (EDSS between 7.5 and 10). Two experienced neurologists evaluated the EDSS scores at nadir and follow-up and also reviewed the medical records to confirm symptoms or signs explainable exclusively by myelopathy, especially in MRI-negative patients. In cases where disagreement was present, consensus was reached after discussion.

### Statistical analysis

Continuous data with a parametrical distribution were expressed as mean and standard deviation (SD) and were compared using Student’s t test, while variables with a nonparametric distribution were expressed as median ± interquartile range (IQR) and compared using the Mann Whitney U test. Categorical data were expressed as numbers (percentages, %), and compared using the chi-square test or Fisher’s exact test. Univariate logistic regression and multivariate logistic regression analyses were performed in sequence to determine the independent predictive factors for IATM patients with a poor prognosis. The level of significance for the two-tailed statistical tests was set at a *p* value < 0.05. The statistical analyses were performed using IBM SPSS version 27.0.1 (IBM, Armonk, NY, USA), while the figures were generated using GraphPad Prism version 8.0.2 (GraphPad Software, San Diego, CA, USA).

## Results

### Detailed selection data

A total of 107 patients with IATM who were hospitalized from May 2018 to May 2022 were screened according to the inclusion and exclusion criteria. Of these patients, 47 were excluded for the following reasons: time from onset to nadir >21 days (*n* = 21), incomplete clinical data (*n* = 6), missing follow-up data (*n* = 3), no first attack (*n* = 14), and severe cardiac, hepatic, or renal insufficiency that may have affected the laboratory tests (*n* = 3). The remaining 132 patients met the criteria for ATM. A further 25 patients with definite etiologies were also excluded: AQP4-IgG-associated (*n* = 4), MOG-IgG-associated (*n* = 3), GFAP-IgG-associated (*n* = 3), CIS (*n* = 3), infectious conditions [tuberculosis (*n* = 3), HSV (*n* = 1), and syphilis (*n* = 1)], surgical injury (*n* = 1), spinal ischemia (*n* = 1), tumor (*n* = 1), compression (*n* = 2) and vitamin B12 deficiency (*n* = 2). The remaining 107 patients were diagnosed with IATM and were considered eligible for the final analysis, with 43 being MRI-negative and 64 being MRI-positive. At the end of follow-up, 80 patients were classified in the good prognosis group and 27 in the poor prognosis group.

### Clinical characteristics and laboratory characteristics of ITAM according to radiographic features

**Fig. 2:**
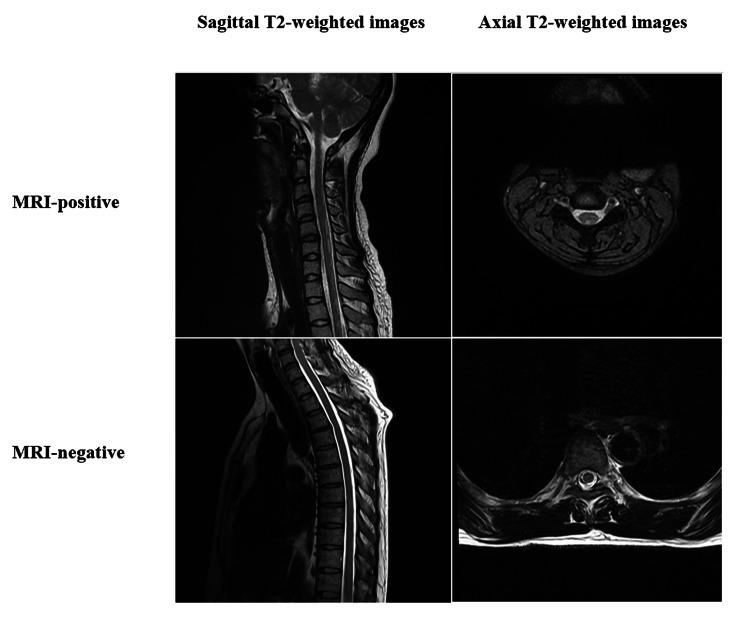
Sagittal/Axial T2-weighted images showing representative examples of spinal cord MRI of patients with MRI-negative and MRI-positive IATM.

Figure 2 shows sagittal and axial T2-weighted images that are representative examples of spinal cord MRI of patients with MRI-negative and MRI-positive IATM. Table 1 summarizes the demographics, clinical characteristics, treatments, prognosis and relapse of all the IATM patients and the comparison between the MRI-negative and MRI-positive groups. Of the 107 IATM patients who eventually met the inclusion criteria, 68 patients use enhanced MRI scanning while routine scanning were performed in the rest 39 patients, finally 64 (59.8%) were identified as MRI-positive, with abnormal signals observed at the cervical level in 33 patients (51.6%), the thoracic level in 34 (53.1%), the lumbar level in 5 (7.8%), and the sacral level in 4 (6.3%). Moreover, among the initial 43 patients in MRI-negative group, 26 patients had repeated spinal cord MRI during their hospital stays and 6 patients turned out lesions. In the whole study cohort, 63 (58.9%) were female, with a median age at onset of 51 years (IQR 39–62 years). The interval between the onset of symptoms and first MRI was 6 days (IQR 3–11 days), while the median time from onset to maximum deficit was 5 days (IQR 2–10 days). A history of a preceding infection or immunization was reported by 15 patients (14.0%). The most common clinical symptoms of neurological dysfunction were sensory disturbance 96 (89.7%), limb weakness 74 (69.2%), sphincter dysfunction 47 (43.9%), and back/neck pain 23 (21.5%). The presence of back/neck pain was significantly more frequent (*P*<0.05) in the MRI-positive group (*n* = 19, 29.7%) compared to that in the MRI-negative group (*n* = 4, 9.3%). Similarly, spinal shock was observed more frequently in patients with an MRI-positive result (*P*<0.05). Signs of upper motor neuron injury were present in 40 patients (37.4%). Regarding treatments, 29 patients (27.1%) had undergone second-line therapies such as PLEX or IVIg. The median length of hospital stay for all 107 patients was 15 days (IQR 11 ~ 19 days), with 2 (1.9%) being admitted to the ICU and 24 (22.4%) developing a complication. With regard to the clinical course over a median follow-up duration of 33 months (IQR 19–47 months), 92 patients (86.0%) remained monophasic, while 15 (14.0%) suffered relapses. Among the 15 patients who had relapses, 2 patients showed a negative MRI and 3 patients found no inflammation in the CSF at the second admission. They all re-checked demyelinating antibodies and OBs, none of them turns into recurrent demyelinating disease associated with antibodies such as MS or NMOSD during the follow-up. A total of 27 IATM patients (25.2%) had a poor prognosis, which was significantly more frequent (*P*<0.01) in those with a positive MRI (34.4%) than in the negative MRI group (11.6%). The median EDSS score at nadir was 4.5 (IQR 2–8) and at follow-up was 1.5 (IQR 1–4). There was no significant difference in the median EDSS score between the two groups, although the median EDSS at follow-up was significantly lower (*P*<0.01) in the MRI-negative patients compared to that in the MRI-positive patients. The laboratory examinations of the two subgroups according to spinal cord MRI in IATM patients is shown in Table 2. Laboratory examinations did not differ significantly between these two groups. All the 107 CSF findings included protein > 450 mg/L in 41 (38.3%), WBC > 10 cells/µL in 15 (14.0%), OB positive in 5 (4.7%), and IgG index > 0.7 in 10 (9.3%).

**Table 1:** Comparisons of demographics, clinical characteristics, treatments, prognosis and relapse between the MRI-negative and MRI-positive IATM patients

	All patients (*n*=107)	MRI-negative (*n*=43)	MRI-positive (*n*=64)	*P* value
Age at onset (years)	51(39~62)	53(42~64)	49.5(32.5~61.8)	0.332
Sex, female	63(58.9%)	22(51.2%)	41(64.1%)	0.184
Time from onset to first MRI (days)	6(3~11)	7(3~10)	6(2~12)	0.669
Time from onset to maximum deficit (days)	5(2~10)	6(3~10)	4(2~10)	0.230
Preceding infection or immunization	15(14.0%)	6(14.0%)	9(14.1%)	0.987
Back/neck pain	23(21.5%)	4(9.3%)	19(29.7%)	0.012*
Sensory disturbance	96(89.7%)	36(83.7%)	60(93.8%)	0.177
Limb weakness	74(69.2%)	27(62.8%)	47(73.4%)	0.242
Sphincter dysfunction	47(43.9%)	17(39.5%)	30(46.9%)	0.453
^a^Signs of upper motor neuron injury	40(37.4%)	16(37.2%)	24(37.5%)	0.976
Spinal shock	36(33.6%)	9(20.9%)	27(42.2%)	0.023*
Second-line therapies(PLEX or IVIg)	29(27.1%)	9(20.9%)	20(31.3%)	0.239
Length of hospital stays (days)	15(11~19)	13(10~19)	15(11.25~19.75)	0.510
Follow-up duration (months)	33(19~47)	35(21~49)	32(19.00~45.25)	0.446
ICU admission	2(1.9%)	0(0.0%)	2(3.1%)	0.515
^b^Complication	24(22.4%)	10(23.3%)	14(21.9%)	0.867
Poor prognosis	27(25.2%)	5(11.6%)	22(34.4%)	0.008*
Relapse	15(14.0%)	6(14.0%)	9(14.1%)	0.987
EDSS at nadir	4.5(2~8)	4.5(2.0~7.5)	4.75(2.13~8.50)	0.290
EDSS at follow-up	1.5(1~4)	1(1~2)	2(1~5)	0.001*
Location of the lesion				
Cervical			33(51.6%)	
Thoracic			34(53.1%)	
Lumbar			5(7.8%)	
Sacral			4(6.3%)	

*P < 0.05. Datas are presented as mean ? standard deviation, number (percentage), or median (interquartile range). PLEX, plasma exchange; IVIg, intravenous immunoglobulin; ICU, intensive care unit; EDSS, expanded disability status scale; MRI, magnetic resonance imaging; ^a^Signs of upper motor neuron injury=(spasticity, hyperreflexia, or positive babinski); ^b^Complication=(Pulmonary infection, deep venous thrombosis of lower limb, pressure ulcer, neurogenic bladder, urinary tract infection).

**Table 2:** Laboratory examinations between the MRI-negative and MRI-positive IATM patients

	All patients (*n*=107)	MRI-negative (*n*=43)	MRI-positive (*n*=64)	*P* value
D-dimer (mg/L)	0.31(0.15~0.88)	0.36(0.13~0.97)	0.26(0.15~0.76)	0.698
WBC counts (×10^9^/L)	7.47(5.78~9.02)	7.59(5.74~9.15)	7.08(5.82~9.00)	0.627
RBC counts (×10^12^/L)	4.34±0.50	4.35±0.51	4.33±0.50	0.883
PLT counts (×10^9^/L)	226±65	234±73	221±59	0.310
NLR	3.52(2.29~5.91)	3.71(2.48~5.56)	3.40(2.00~6.95)	0.691
Albumin (g/L)	40.6±4.2	40.7±4.0	40.6±4.4	0.986
CK (U/L)	74(49~113)	82(57~117)	72(45.7~110.5)	0.423
Blood potassium (mmol/L)	3.8±0.4	3.9±0.4	3.8±0.3	0.486
Blood sodium (mmol/L)	140±3	140±3	140±3	0.413
CSF				
Protein >450 mg/L	41(38.3%)	18(41.9%)	23(35.9%)	0.537
WBC >10 cells/μL	15(14.0%)	6(14.0%)	9(14.1%)	0.987
OB positive	5(4.7%)	0(0.0%)	5(7.8%)	0.159
IgG index >0.7	10(9.3%)	3(7.0%)	7(10.9%)	0.703

*P < 0.05. Datas are presented as mean ± standard deviation, number (percentage), or median (interquartile range). WBC, white blood cells; RBC, red blood cells; PLT, platelet; NLR, neutrophil-to-lymphocyte ratio; ALT, alanine aminotransferase; AST, aspartate aminotransferase; CK, creatine kinase; CSF, cerebrospinal fluid; OB, oligoclonal bands

### Clinical characteristics and laboratory characteristics of IATM according to their outcome

Comparisons of the demographics, clinical characteristics, treatments, prognosis and relapse of patients with IATM grouped according to clinical outcome are shown in Table 3. Of the 107 IATM patients included in the study, 27 (25.2%) had a poor prognosis at the last follow-up. Comparison of patients with either a good or poor prognosis showed that there was no statistically significant difference in gender or age between the two groups at the onset of the disease. However, we observed a statistically significant difference in the time from onset to first MRI between the patients with a good prognosis compared to those with a poor prognosis (7.5 days vs. 3 days, respectively, *P*<0.01), whereas there was no difference in the time from onset to maximum deficit between the two groups. The incidence of limb weakness (*P*<0.01), and sphincter dysfunction (*P*<0.01) was significantly higher in the poor prognosis group than in the good prognosis group. However, no statistically significant differences were observed between the two groups for preceding infection, immunization, back or neck pain, sensory disturbances, signs of upper motor neuron injury, and spinal shock. The proportion of patients who received second-line therapies (i.e., PLEX or IVIg)was significantly higher in patients with a poor prognosis than those with a good prognosis (63% vs. 15%, respectively, *P*<0.01). Patients with a poor prognosis had a significantly longer (*P*<0.01) median length of hospital stay compared to those with a good prognosis (18 days [IQR 13–25] vs. 13 days [IQR 10-17.8], respectively). No statistical difference was observed between the two groups for the occurrence of an ICU admission, complications, or a relapse. With a similar duration of follow-up, the EDSS at nadir (*P*<0.01) and follow-up (*P*<0.01) both tended to be higher in the poor prognosis group than in the good prognosis group, with the differences being markedly significant. Of note, a positive MRI result was observed more frequently in patients with a poor prognosis compared to those with a good prognosis (81.5% vs. 52.5%, respectively, *P*<0.01). Regarding the laboratory examinations, patients with a poor prognosis had higher WBC counts (*P*<0.01) and NLR (*P*<0.01) than those with a good prognosis (Table 4).

**Table 3:** Demographics, clinical characteristics, treatments, prognosis, and relapse of patients with IATM grouped according to their clinical outcome

	Good prognosis (*n*=80)	Poor prognosis (*n*=27)	*P* value
Age at onset (years)	53.5(41~62)	43(31~65)	0.443
Sex, female	47(58.8%)	16(59.3%)	0.963
Time from onset to first MRI (days)	7.5(3~12)	3(1~9)	0.008*
Time from onset to maximum deficit (days)	5(2.3~10.0)	3(2~12)	0.724
Preceding infection or immunization	11(13.8%)	4(14.8%)	1.000
Back/neck pain	15(18.8%)	8(29.6%)	0.234
Sensory disturbance	72(90.0%)	24(88.9%)	1.000
Limb weakness	48(60.0%)	26(96.3%)	<0.001*
Sphincter dysfunction	29(36.3%)	18(66.7%)	0.006*
Signs of upper motor neuron injury	29(36.3%)	11(40.7%)	0.677
Spinal shock	23(28.8%)	13(48.1%)	0.065
Second-line therapies(PLEX or IVIg)	12(15.0%)	17(63.0%)	<0.001*
Length of hospital stays (days)	13(10~17.8)	18(13~25)	0.004*
Follow-up duration (months)	32(18~47)	36(22~48)	0.362
ICU admission	0(0.0%)	2(7.4%)	0.062
Complication	15(18.8%)	9(33.3%)	0.116
Relapse	10(12.5%)	5(18.5%)	0.647
EDSS at nadir	3.5(2~7)	8.5(6.5~9.0)	<0.001*
EDSS at follow-up	1(1~2)	5(5~7)	<0.001*
MRI-positive	42(52.5%)	22(81.5%)	0.008*

*P < 0.05. Datas are presented as mean ± standard deviation, number (percentage), or median (interquartile range). PLEX, plasma exchange; IVIg, intravenous immunoglobulin; ICU, intensive care unit; EDSS, expanded disability status scale; MRI, magnetic resonance imaging; ^a^Signs of upper motor neuron injury=(spasticity, hyperreflexia, or positive babinski); ^b^Complication=(Pulmonary infection, deep venous thrombosis of lower limb, pressure ulcer, neurogenic bladder, urinary tract infection)

**Table 4:** Comparison of laboratory examinations of patients with IATM with either a good or poor prognosis

	Good prognosis (*n*=80)	Poor prognosis (*n*=27)	*P* value
D-dimer (mg/L)	0.27(0.13~0.82)	0.48(0.19~1.12)	0.179
WBC counts (×10^9^/L)	6.92(5.66~8.27)	8.95(7.07~12.58)	**0.001** ^*^
RBC counts (×10^12^/L)	4.30±0.51	4.45±0.46	0.845
PLT counts (×10^9^/L)	224±63	224±72	0.486
NLR	3.18(2.00~5.15)	4.39(3.52~8.81)	**0.004** ^*^
Albumin (g/L)	40.6±4.0	40.7±4.9	0.924
CK (U/L)	71(48~102)	92(51~130)	0.231
Blood potassium (mmol/L)	3.8±0.4	3.9±0.4	0.306
Blood sodium (mmol/L)	140±3	139±3	0.071
CSF			
Protein >450 mg/L	32(40.0%)	9(33.3%)	0.538
WBC >10 cells/μL	11(13.8%)	4(14.8%)	1.000
OB positive	3(3.8%)	2(7.4%)	0.802
IgG index >0.7	6(7.5%)	4(14.8%)	0.907

*P < 0.05. Datas are presented as mean ± standard deviation, number (percentage), or median (interquartile range). WBC, white blood cells; RBC, red blood cells; PLT, platelet; NLR, neutrophil-to-lymphocyte ratio; CK, creatine kinase; CSF, cerebrospinal fluid; OB, oligoclonal bands,

### Multivariate logistic regression analysis of predictive factors for poor prognosis

The multivariate logistic regression analysis showed the following predictive factors were associated independently with a poor outcome (Table 5): absence of second-line therapies (OR = 0.155; 95% CI = 0.037–0.657; *P*<0.05), high EDSS score at nadir (OR = 1.448; 95% CI = 1.038–2.019; *P*<0.05), and a positive MRI result (OR = 0.118; 95% CI = 0.026–0.535; *P*<0.05).

**Table 5:** Multivariate logistic regression analysis of predictive factors for poor prognosis in IATM patients

Variables	Multivariate analysis				
	Basic model			Adjusted model	
	OR (95% CI)	*P* value		OR (95% CI)	*P* value
Age at onset (years)				0.969(0.932~1.007)	0.104
Sex, female				0.443(0.113~1.734)	0.242
Limb weakness	0.137(0.011~1.716)	0.123		0.170(0.014~2.028)	0.161
Sphincter dysfunction	0.755(0.206~2.764)	0.672		0.734(0.192~2.802)	0.651
Second-line therapies (PLEX or IVIg)	0.202(0.052~0.779)	0.020*		0.155(0.037~0.657)	0.011*
Length of hospital stays (days)	1.009(0.936~1.087)	0.820		1.015(0.942~1.093)	0.705
EDSS at nadir	1.354(0.999~1.835)	0.051		1.448(1.038~2.019)	0.029*
MRI-positive	0.171(0.043~0.676)	0.012*		0.118(0.026~0.535)	0.006*
WBC counts (×10^9^/L)	1.192(0.968~1.467)	0.099		1.209(0.972~1.504)	0.089
NLR	0.975(0.899~1.057)	0.539		0.983(0.913~1.059)	0.656

OR, odds ratio; CI, confidence interval

### Comparison of the EDSS scores for ITAM at nadir and follow-up

**Fig. 3:**
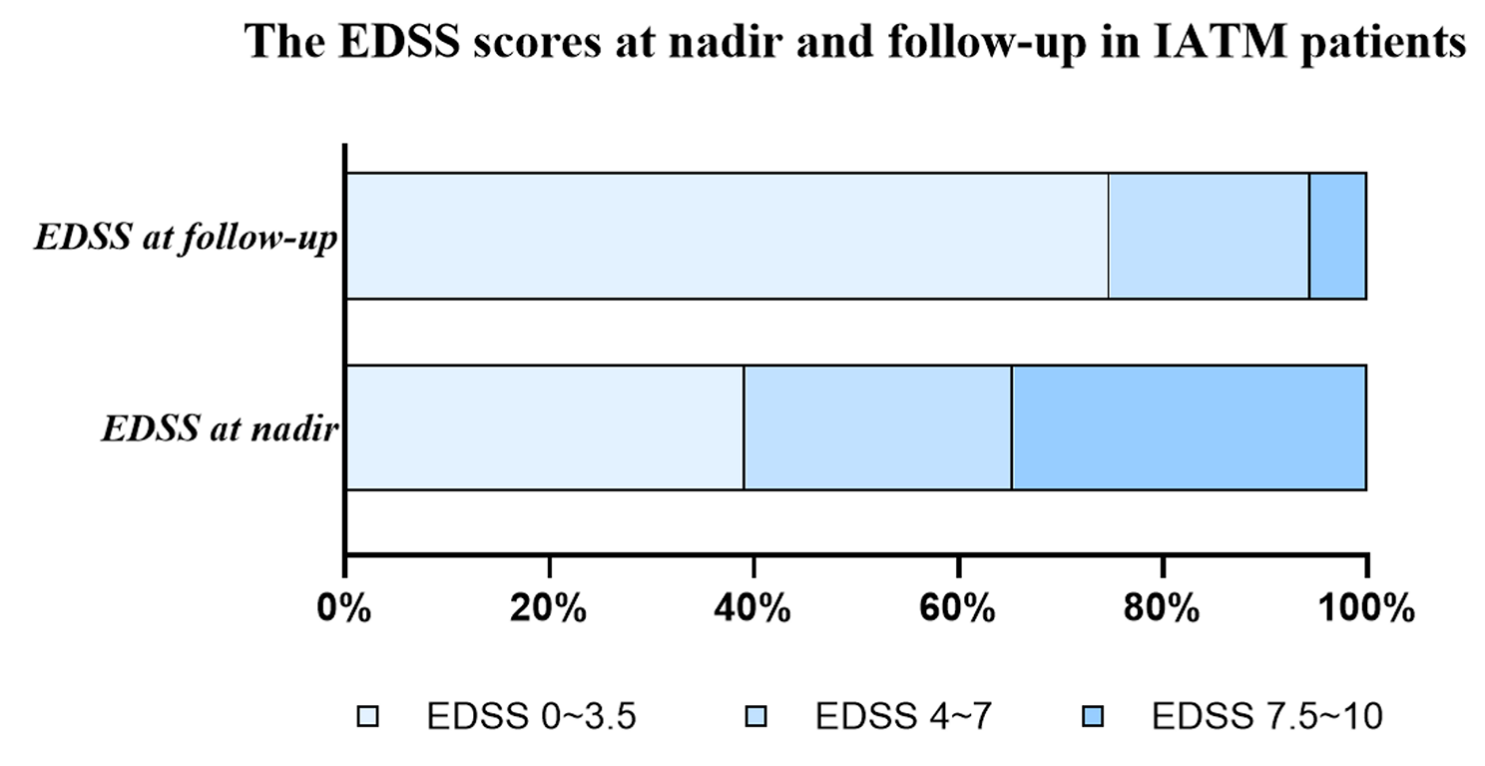
Contrast in EDSS scores at nadir and follow-up

Figure 3 shows the comparison of the EDSS scores at nadir and follow-up. In the nadir neurological evaluation, the median EDSS score was 4.5 (IQR 2–8), with three distinct groups being defined: mild disability (39.2%), moderate disability (26.2%), and severe disability (34.6%). In comparison, the median EDSS score at follow-up was 1.5 (IQR 1–4) with the groups divided as above, showing mild disability (74.8%), moderate disability (19.6%), and severe disability (5.6%).

## Discussion

It has been reported previously that IATM results in motor, sensory, and autonomic dysfunction and is associated with a poor prognosis. However, the comparative analysis of MRI-negative and MRI-positive in patients with first-attack IATM were rarely reported. Therefore, we selected a relatively large cohort that met the criteria for IATM established by the TMCWG, which involved comparing the clinical characteristics, demographics, laboratory examinations, treatments, prognosis and relapse between patients with MRI negative or positive results. We also investigated the factors that predicted a poor prognosis and considered that such a study would be particularly vital for early diagnosis of IATM and for making individualized therapeutic regimens that would improve the prognosis of patients with the disorder.

Previous studies in multiple centers and using various episodes have reported that the prevalence of IATM in TM ranges between 6 and 59% [2, 9–10], similar to the rate of 107/179 (60%) we observed in our study. The clinical outcomes in our patient cohort were also very similar to those reported previously [2, 11]. We showed there were no gender disparities between male and female patients with IATM, with only a slight predominance towards females. A proportion of IATM patients may have had a preceding infection or immunization [1, 12], with our study showing this frequency was 14%. In addition, 64 patients in our study had abnormal spinal cord MRI scans, with six of these patients having normal neuroimaging studies at the first MRI, whereas their repeat MRIs were positive. Therefore, a repeat MRI after first negative-MRI is necessary in uncertain cases to avoid missing the diagnosis of myelitis. Previous studies in IATM have reported a rate for normal spinal MRIs of 5% [13],10% [14], 22% [6] and 50% [15]. However, the reason why patients with IATM have a negative MRI remains uncertain but we speculate this may occur for the following reasons. Firstly, the inflammatory lesions in the spinal cord may be mild and therefore the blood-brain barrier may not have been breached. Secondly, the time of the imaging was not correct, with early imaging missing a progressive lesion, or late imaging omitting a transient lesion. Thirdly, the magnetic field was not sufficiently sensitive to identify lesions, or certain imaging sequences were immature [16–17]. Therefore we consider it important that more detailed basic research on MRI-negative IATM is carried out in the future.

Of note, we showed that MRI-negative IATM patients were more likely to have a good prognosis compared to that in the MRI-positive group, and also had a significantly lower EDSS at follow-up. This finding may be explained by less severe lesions in MRI-negative patients. However, there are discrepancies in the magnetic field of MRI scanners and also in the imaging methods used in our study due to its retrospective nature. It is now widely accepted that 3.0 T scanners or gadolinium enhanced imaging are better for visualizing lesions [16, 18], with new MRI sequences such as double inversion recovery or phase sensitive inversion recovery more sensitive for revealing spinal cord lesions [19]. There is also evidence that diffusion tensor imaging can identify secret lesions and has potential in the future to assess prognosis, although its clinical application may be limited due to technical difficulties [18, 20]. It is worth noting that there were no significant differences in EDSS at nadir between the MRI-positive and -negative groups, which indicated that spinal cord MRI may be negative in patients with IATM despite them having severe manifestations. The results of our study indicated that the presence of back/neck pain and spinal shock was more frequent in the MRI-positive group than in the MRI-negative group. This suggests that IATM patients with a positive MRI are not necessarily more sensitive in terms of clinical symptoms compared to patients with a negative MRI. However, larger cohort studies are required to validate this possibility given the discrepancies we observed between the two groups. In conclusion, a low EDSS score at follow-up was shown to be the only prognostic factor for a MRI-negative IATM.

Fifteen (14.0%) of our cohort experienced a recurrence of IATM, although none of these patients converted to recurrent demyelination associated with antibodies such as MS or NMOSD during the study period. It has been reported that the frequency of recurrences varies between 17%~57% [2, 21–24] in IATM patients during long-term follow-up, which is higher than the percentage observed in our patient cohort. Previous studies have reported that 0%~29% [6, 13, 22–23, 25–26] of IATM patients subsequently converted to MS over a mean follow-up period ranging from 2 to 8 years. Although the median follow-up period of 33 months in our study was long enough to capture a recurrent course and adverse clinical outcome, a longer follow-up may not only increase the frequency of relapse and better prognosis in the future, but also identify patients with recurrent demyelination associated with antibodies [2, 27]. Long-term neurological follow-up in the current study demonstrated that the majority (74.8%) of IATM patients had a favorable prognosis. The incidence of a poor prognosis was similar in our study to that reported by other studies on IATM that ranged between 11 and 38.9% [21–22, 28, 29, 30]. Numerous factors associated with a poor prognosis have been described in patients with IATM, including back pain [31], a high deficit score at onset [32], a high mRS score on admission [28], time to maximal deficit within 24 h [21], spinal shock [22], urinary sphincter dysfunction [21, 33], and longitudinally extending transverse myelitis (LETM) [23, 33]. Our study showed that factors associated with a poor prognosis included an absence of second-line therapies, a high EDSS score at nadir, and a positive MRI. Our results also indicated that more aggressive therapies (intravenous corticosteroids combine with IVIg or PLEX) should be administered when these predictors are identified. A lower EDSS at nadir, less time from onset to first MRI, and a lower rate of a positive MRI in the group with a good prognosis could be explained by these patients having milder condition. Therefore further studies are needed to determine the relationship between a negative MRI and a poor prognosis in IATM patients. A previous study [22] reported that the loss or a decrease in spinal cord reflexes below the level of the injury suggesting spinal shock [34] was the only risk factor for a poor outcome at the end of follow-up. However, we observed no correlation between spinal shock and prognosis in our patient cohort. We also demonstrated that second-line therapies including PLEX or IVIg were related significantly to a good prognosis, a finding in agreement with that reported by Greenberg [35]. As for the mechanisms involved, Lehmann suggested that PLEX could clear autoantibodies in the blood circulation, immune complexes, cytokines and other inflammatory mediators, while IVIg played a role in immunomodulatory by the neutralization of autoantibodies, the elimination of activated complement, the alteration of FcR and cytokine patterns [36]. The WBC counts and NLR measured in our study were also significantly higher in patients with a poor outcome compared with those with a good prognosis, which indicated these patients had a more severe inflammatory reaction. Nevertheless, these two factors did not predict a poor outcome. Our study showed that the incidence of CSF OB positivity was 4.7% in IATM patients, while relevant data from several previous studies showed this rate ranged between 0%~17% [22, 30]. As reported in our previous study [22], we did not find any difference between a good or poor prognosis in terms of CSF parameters. Other studies have emphasized that the presence of OCB in the CSF is associated with a higher risk of developing MS [37, 38]. Moreover, in the five IATM patients in the current study whose CSF OB was positive, none converted to MS over a median follow-up of 33 months. However, further larger studies are needed to validate this absence of conversions. There are also conflicting results about the predictive value of CSF pleocytosis. In some studies, CSF pleocytosis was associated with a poor prognosis [13, 39], whereas in others it did not affect clinical outcome [30, 31]. In our study, CSF pleocytosis appeared to have no effect on clinical outcome. These discrepancies between studies may be explained by the timing of CSF sampling and the different mean duration of follow-up [40]. Previous studies have suggested that the 14-3-3 protein [41], IL-6 [42] and non-specific enolase [43] in the CSF are inflammatory markers for tissue injury and sustained clinical disability and serve as helpful prognostic indicators in ATM. However, we could not investigate these factors due to the retrospective nature of our study.

Firstly, the number of cases was relatively small, and therefore the reliability of the results needs to be validated in a prospective study including more cases. Secondly, all the patients were from one single center and the follow-up period for each patient was inadequate. Therefore, the results of this study require validation in multicenter and prospective studies with longer follow-up periods.

## Conclusion

In summary, this study raises the awareness that a low EDSS score at follow-up may be associated with a negative MRI result in patients with first-attack IATM. The absence of second-line therapies, a high EDSS score at nadir, and a positive MRI are risk factors for a poor prognosis. Early recognition of this possible MRI-negative IATM is important because second-line immunotherapy may improve clinical outcome.

## Data Availability

All relevant data are described within the paper. Deidentified data can be requested. Data can be requested by all interested researchers, who can be contacted via the corresponding author.

## Abbreviations

IATM: Idiopathic acute transverse myelitis
MS: Multiple sclerosis
NMOSD: Neuromyelitis optica spectrum disorders
ADEM: Acute disseminated encephalomyelitis
CIS: Clinically isolated syndrome
MOGAD: Myelin oligodendrocyte glycoprotein associated disorders
GFAP: Glial fibrillary acidic protein astrocytopathy
CSF: Cerebrospinal fluid
MRI: Magnetic resonance imaging
OB: Oligoclonal bands
TMCWG: Transverse Myelitis Consortium Working Group
AQP4-IgG: Aquaporin-4 immunoglobulin G
ICU: Intensive care unit
IVIg: Intravenous immunoglobulins
PLEX: Plasma exchange
CNS: Central nervous system
EDSS: Expanded Disability Status Scale
SD: Standard deviation
IQR: Interquartile range
LETM: Longitudinally extending transverse myelitis
